# Specialized infrastructure in a tertiary hospital to administer disease modifying treatments in Alzheimer’s disease

**DOI:** 10.1002/alz.086758

**Published:** 2025-01-09

**Authors:** Talya Nathan, Elissa Ash, Dror Shir, Noa Trabulus, Aya Bar‐David, Galia Wolpe, Tamara Shiner, Noa Bregman

**Affiliations:** ^1^ Tel Aviv Sourasky Medical Center, Tel Aviv Israel; ^2^ Sackler School of Medicine, Tel Aviv University, Tel Aviv Israel; ^3^ Sagol School of Neuroscience, Tel Aviv University, Tel Aviv Israel

## Abstract

**Introduction:**

Lecanemab (LEQEMBI®), a humanized monoclonal antibody targeting Amyloid‐beta (Aβ) protofibrils, received full FDA approval in July 2023 for treating early‐stage Alzheimer’s disease (AD). This abstract highlights Tel Aviv Medical Center’s (TLVMC) specialized infrastructure for early AD diagnosis and treatment and includes presenting baseline characteristics of initial patients opting for LEQEMBI®.

**Methods:**

Outlining our clinics' operational experience in establishing the Center for advanced treatments for AD, treatment protocol, and a descriptive analysis of baseline assessment data including demographics, baseline Magnetic‐Resonance‐Imaging (MRI), Cerebrospinal‐fluid (CSF)/PET biomarkers, pre‐treatment cognitive evaluations (Mini‐Mental‐State‐Examination (MMSE)/Montreal‐Cognitive‐Assessment (MoCA)), and Apolipoprotein‐E (APOE) status.

**Results:**

Rigorous screening and specialist approval were prerequisites for candidate selection, adhering with published appropriate use recommendations. All patients, diagnosed with Mild‐Cognitive‐Impairment (MCI)/mild dementia due to AD confirmed by biomarkers, underwent APOE testing.

Lecanemab Administration commenced in Israel in November 2023, with 31 patients (F = 15, mean age = 72) initiating treatment by year‐end. 22 underwent neurological evaluation within a year of symptom onset. Mean MMSE = 24 (n = 27, SD = 3) mean MoCA = 23 (n = 4, SD = 2). CSF testing (27 patients), showed mean levels (pg/ml) of total‐Tau = 585 (SD = 251), phosphorylated‐Tau = 121 (SD = 38) and Aβ = 261 (SD = 172). APOE Ɛ4 polymorphism was present in 55%, with 18% showing 1‐2 micro‐hemorrhages on baseline MRI, compared to 8% of non‐carriers.

**Conclusion:**

The evolving landscape of AD treatments necessitates complex screening and comprehensive clinical‐radiological follow‐up. Establishing specialized medical infrastructures is crucial to enable the wide and safe administration of new treatments, reflecting the ongoing paradigm shift in AD management.

